# Case Report: Minimally invasive surgical management of a complex high rectovaginal fistula caused by long-term retained and migrated intrauterine device

**DOI:** 10.3389/fmed.2026.1863833

**Published:** 2026-06-24

**Authors:** Ziwen Wang, Zhiyin Deng, Min Zhu, Li Zhang, Haixia Xu, Zhen Zhou, Yanqing Lu, Hao Wang

**Affiliations:** 1 Department of Coloproctology, The Affiliated Guangzhou Hospital of TCM of Guangzhou University of Chinese Medicine, Guangzhou, China; 2 Department of Gynecology, The Second Affiliated Hospital of Guangzhou University of Chinese Medicine, Guangzhou, China; 3 Department of Colorectal Surgery, The Second Affiliated Hospital of Guangzhou University of Chinese Medicine, Guangzhou, China; 4 Guangzhou University of Chinese Medicine, Guangzhou, China

**Keywords:** case report, intrauterine device migration, laparoscopy, minimally invasive surgery, multidisciplinary team, rectovaginal fistula

## Abstract

**Background:**

Rectovaginal fistula (RVF) secondary to migrated intrauterine device (IUD) is an extremely rare complication, and high-level RVF with multiple prior failed IUD removal attempts remains a formidable clinical challenge.

**Case presentation:**

We report a 43-year-old female who presented with vaginal fecal discharge for 3 weeks. She underwent insertion of an oval metallic IUD in 2014, which was confirmed to have migrated during induced abortion, and had a failed laparoscopic-hysteroscopic IUD removal attempt in 2022. During that attempt, intraoperative findings revealed the IUD had migrated to the left pelvic floor; however, the procedure was terminated due to concerns regarding the complex surrounding tissue structure and the surgeon’s technical limitations at a smaller-scale hospital, as the patient was not experiencing significant physical discomfort at that time. Preoperative examinations revealed a 1-cm fistula at the posterior vaginal fornix and an annular metallic IUD embedded in the anterior rectal wall 8 cm from the anal verge. After multidisciplinary consultation, the patient underwent laparoscopic proctectomy, combined transabdominal-transvaginal rectovaginal fistula repair, and temporary ileostomy. The operation was uneventful, and no postoperative complications occurred. Ileostomy closure was performed at 3 months postoperatively, and no fistula recurrence, fecal incontinence, or other adverse events were observed during the 6-month follow-up.

**Conclusion:**

The combined laparoscopic transabdominal-transvaginal approach is a safe and effective surgical option for high-level RVF secondary to migrated IUD, offering the advantages of precise exposure, minimal trauma, and low recurrence risk. This case highlights the importance of long-term follow-up after IUD insertion and the value of multidisciplinary collaboration in managing complex pelvic fistulas.

## 1 Introduction

RVF is an abnormal epithelialized tract between the rectum and vagina, characterized by vaginal passage of flatus or feces, a condition that causes severe physical and psychosocial distress (1). High-level RVF are defined as abnormal fistulous tracts formed between the middle third of the rectum and the posterior vaginal fornix (2), and this condition remains a formidable clinical challenge due to the inherent difficulties in surgical exposure, a high postoperative recurrence rate, and a non-negligible risk of adjacent organ injury during intervention (3). The IUD, being a reversible and effective contraceptive method, is the most widely used contraceptive method worldwide (4). High-level RVF secondary to a migrated IUD is an extremely rare complication, with few cases reported globally, especially in patients with prior failed IUD removal attempts (5).

A literature search was performed using databases including PubMed, MEDLINE, Web of Science, and others. The search terms included (“intrauterine device” OR “IUD”) AND (“rectovaginal fistula” OR “rectal migration”). A thorough review of the literature confirmed the rarity of this condition; while isolated cases of IUD migration to the rectum exist, this is indeed the first documented case of a high-level RVF caused by a long-term retained migrated metallic IUD with a prior failed removal attempt. To our knowledge, this is the first reported case of high-level RVF secondary to a 10-year retained migrated metallic IUD with prior failed laparoscopic-hysteroscopic removal attempts, successfully treated via a combined laparoscopic transabdominal-transvaginal minimally invasive approach. Herein, we describe the clinical course, diagnostic workup, surgical technique, and follow-up outcomes of this rare case and discuss the relevant medical literature to provide clinical guidance for similar cases.

## 2 Case report

A 43-year-old female was admitted with fecal discharge from the vagina for 3 weeks. She underwent insertion of an oval metallic IUD in 2014, and IUD migration was confirmed during induced abortion, and she had a failed laparoscopic-hysteroscopic IUD removal attempt in 2022. During this procedure, the IUD was located in the left pelvic floor. Due to the limited technical capabilities at the facility, concerns regarding the complexity of the surrounding tissue anatomy, and the patient not reporting significant physical discomfort at that time, the operating surgeon decided to terminate the intervention. Her obstetric history was significant for 4 pregnancies and 2 vaginal deliveries (G4P2). She delivered her first child vaginally in 2009 and underwent an induced abortion with curettage in 2011. She delivered her second child vaginally in 2013. The IUD was inserted in 2014. Notably, she experienced an unintended pregnancy with the IUD in place, resulting in a second induced abortion with curettage without IUD removal. She also had a history of chronic pelvic inflammatory disease.

**Figure 1:**
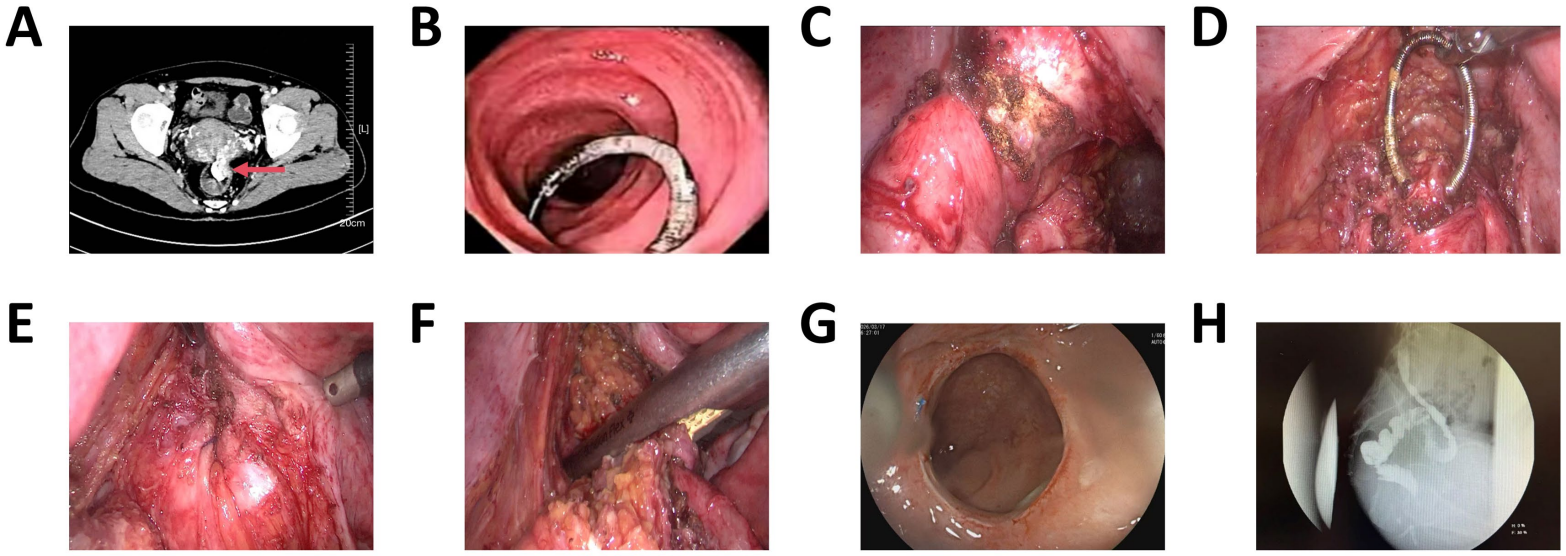
Clinical, intraoperative and postoperative imaging data of the patient with IUD-related complex high rectovaginal fistula. **(A)** Preoperative pelvic contrast-enhanced computed tomography, with an arrow pointing to the migrated metallic IUD embedded in the rectal wall. **(B)** Preoperative colonoscopy showing the annular metallic IUD protruding into the rectal lumen. **(C)** Intraoperative exploration showing the rectovaginal fistula tract at the posterior vaginal fornix. **(D)** Intraoperative photograph of the completely removed oval metallic IUD. **(E)** Intraoperative photograph showing tension-free suturing of the posterior vaginal wall fistula. **(F)** Intraoperative photograph of the resected rectal segment containing the fistula tract and IUD embedding site. **(G)** Postoperative colonoscopy at 3 months, showing well-healed rectal anastomosis with no residual fistula. **(H)** Postoperative defecography at 3 months, confirming no fistula recurrence and normal rectoanal functional morphology.

Physical examination revealed a 1-cm fistula in the posterior vaginal fornix with fecal outflow and a foreign body palpable on the anterior rectal wall 8 cm from the anal verge. Preoperative pelvic contrast-enhanced computed tomography (CT) suggested the presence of a uterorectal fistula and revealed an annular metallic density (Figure 1A), while preoperative colonoscopy clearly showed an annular metallic IUD embedded in the rectal wall (Figure 1B).

Following a multidisciplinary consultation (gynecology and colorectal surgery), the patient underwent laparoscopic proctectomy, combined transabdominal-transvaginal rectovaginal fistula repair, and temporary ileostomy under general anesthesia. Pneumoperitoneum was established, and mobilization of the rectum and sigmoid colon were performed along the avascular Toldt’s plane and rectorectal space, ensuring preservation of the ureters and pelvic autonomic nerves. Dense adhesions between the rectum, uterus, and posterior vaginal fornix were dissected, and the fistula orifice at the posterior vaginal fornix was clearly identified (Figure 1C). The migrated metallic IUD was detected through the fistula tract, and the intact oval metallic IUD was successfully removed (Figure 1D). Subsequently, gynecological surgeons exposed the fistula tract transvaginally and performed a tension-free repair using 3–0 absorbable sutures (Figure 1E). The diseased rectal segment containing the fistula tract and the IUD embedding site was then resected (Figure 1F), and colorectal anastomosis was completed with a circular stapler without opening the fistula tract into the abdominal cavity to avoid contamination. A well-vascularized omentum was mobilized and interposed between the rectum and the vaginal septum, and a temporary ileostomy was performed for fecal diversion.

The operation was uneventful. The operative time was 192 min, with an estimated blood loss of approximately 15 mL. The patient had no postoperative complications. Histopathological examination of the resected rectal segment confirmed chronic inflammatory cell infiltration, foreign body granuloma formation, and the presence of a fistula tract consistent with IUD-induced chronic injury. Postoperative colonoscopy showed a well-healed rectal anastomosis with no residual foreign body or fistula orifice (Figure 1G), and postoperative defecography further confirmed the absence of fistula recurrence with normal rectal and anal functional morphology (Figure 1H). The patient’s postoperative hospital stay was 6 days. Ileostomy closure was performed laparoscopically at 3 months postoperatively, and no fistula recurrence, fecal incontinence, or other adverse events were observed during the 6-month follow-up period.

## 3 Discussion

### 3.1 Strengths and limitations of the approach

The combined laparoscopic transabdominal-transvaginal approach used in this case has several key strengths. First, compared with the combined laparoscopic-hysteroscopic approach alone used in 2022, the combined laparoscopic transabdominal-transvaginal approach adopted in this case provides more comprehensive and three-dimensional exposure of the pelvic floor structures, particularly the posterior vaginal fornix and the deep pelvic spaces where the migrated IUD was located. This technique facilitates meticulous dissection while significantly reducing the risk of iatrogenic injury to adjacent pelvic organs, including the ureters, bladder, and reproductive vessels. Second, direct resection of the fistula-bearing rectal segment without opening the fistula tract into the abdominal cavity effectively prevents intra-abdominal infection, which is the leading cause of failure in conventional RVF repairs (6). Additionally, the minimally invasive laparoscopic approach allows for precise protection of the pelvic autonomic nerve plexuses, reducing the risk of postoperative urinary and sexual dysfunction. Lastly, the integrated approach combining resection of the fistula-bearing intestinal segment resection, tension-free vaginal repair, omental interposition, and temporary fecal diversion collectively creates an optimal healing environment conducive to wound healing.

However, this approach also has some limitations. First, it is technically demanding and requires experienced surgeons in both colorectal and gynecological laparoscopic surgery. Second, it involves resection of a segment of normal rectum, which carries a small risk of anastomotic leakage and stricture formation. Third, although the 6-month follow-up in this case showed no recurrence, this duration is relatively short. We explicitly acknowledge that longer-term follow-up is necessary to fully assess the risk of late recurrence and long-term functional outcomes.

### 3.2 Discussion of relevant medical literature

Complications related to IUD insertion encompass insertion failure, pain, vasovagal reactions, infection, abnormal uterine bleeding, and expulsion (7). Among them, uterine perforation and IUD migration are rare complications with an incidence of approximately 0.1% (8), and the majority of documented cases manifest within 4 years following IUD placement (9). In the present case, the IUD remained *in situ* for more than 10 years, and secondary high-level RVF resulting from its migration is extremely rare. The pathogenesis of IUD-induced high-level RVF in this patient is hypothesized to result from chronic mechanical irritation of the rectal and vaginal walls by the metallic IUD, which provokes sustained inflammation, formation of foreign body granulomas, and ultimately leads to tissue necrosis and fistula tract development (10).

Methods for retrieving an IUD that has perforated into the rectum include endoscopy, laparoscopy, and laparotomy, depending on the device’s location, the degree of embedment in the rectal wall, involvement of other organs, and the presence of concurrent complications (11). In cases where most of the IUD lies within the intestinal lumen and is not firmly embedded in the rectal wall, successful retrieval via colonoscopy has been reported (12). Meanwhile, the combined laparoscopic-colonoscopic approach has been documented to effectively treat isolated rectal perforation caused by IUD migration, but this method is only suitable for early-stage cases without fistula formation, chronic inflammation, or dense pelvic adhesions (13). In contrast, the patient in this case had a migrated IUD retained for 10 years, which had led to the development of a high-level RVF, complicated by one prior failed removal attempt and severe pelvic adhesions. Retrieval was not feasible with laparoscopy alone or in combination with endoscopy, necessitating a novel approach to remove the IUD and repair the resulting high-level RVF.

Currently, several therapeutic options are available for the treatment of RVF. In most patients with low RVF, the first line therapeutic option is an endorectal advancement flap with or without sphincteroplasty (14). Meanwhile, there is also evidence to show that modified single-incision Martius flap transfer (15) and gracilis muscle transplantation have achieved favorable clinical results in patients with RVF (16). However, the optimum surgical approach for high-level RVF, especially high-level RVF secondary to a migrated IUD, remains controversial. Standard surgical procedures include transanal repair, transvaginal repair, and transabdominal repair (17). Nevertheless, transanal and transvaginal approaches frequently offer insufficient visualization of high fistula tracts, thereby complicating the complete excision of the fistula and retrieval of the migrated IUD, especially in patients presenting with dense pelvic adhesions resulting from previous surgical interventions. Although open transabdominal surgery affords superior exposure, it is associated with significant surgical trauma, longer hospital stays, and an increased incidence of complications (18).

In recent years, laparoscopic surgery has been a promising alternative for the treatment of high-level RVF. It has been shown in several studies that laparoscopic repair of RVF has similar success rates to open surgery with the added benefits of less surgical trauma, less postoperative pain, shorter hospital stays, and quicker recovery (19). However, in cases where the fistula tract extends to the posterior vaginal fornix, laparoscopic surgery alone may still be inadequate. This is because it is difficult to obtain adequate exposure and perform a tension-free vaginal repair through the abdominal approach alone. The integrated laparoscopic transabdominal-transvaginal approach employed in this case capitalizes on the benefits inherent to each approach. Specifically, the transabdominal approach allows complete mobilization of the rectum and sigmoid colon, whereas the transvaginal route affords superior visualization of the posterior vaginal fornix, thereby facilitating accurate excision and subsequent repair of the fistulous tract.

### 3.3 Take-away lessons

This case underscores several important clinical lessons. Firstly, long-term follow-up is essential after IUD insertion, especially in patients with confirmed IUD migration. Prompt identification and extraction of migrated IUDs are vital to avert severe complications, including fistula development. Secondly, in patients with prior failed IUD removal attempts, a multidisciplinary approach involving colorectal surgeons and gynecologists is crucial to plan the optimal surgical strategy. Thirdly, the combined laparoscopic transabdominal-transvaginal approach is a safe and effective option for the treatment of complex high-level RVF secondary to a migrated IUD, offering precise exposure, minimal trauma, and low recurrence risk. Lastly the implementation of temporary fecal diversion alongside omental interposition serves as a valuable adjunctive strategy to enhance the success rates of high-level RVF repairs in patients considered high-risk cases.

## 4 Summary

In conclusion, the combined laparoscopic transabdominal and transvaginal approach is a safe and effective surgical option for the treatment of high-level RVF secondary to a migrated IUD. It offers the advantages of minimal surgical trauma, precise surgical exposure, and low postoperative recurrence risk in the management of patients with high-level RVF. However, longer follow-up is needed to fully assess recurrence and functional outcomes. Given the few published reports on this surgical technique to date, further studies with larger sample sizes and randomized controlled trials are warranted to validate its efficacy and long-term clinical safety.

## Data Availability

The original contributions presented in the study are included in the article/supplementary material, further inquiries can be directed to the corresponding author.
